# Supplementary material for: “Shedding Light on Light”: A Review on the Effects on Mental Health of Exposure to Optical Radiation
Source: Int J Environ Res Public Health. 2021 Feb 9;18(4):1670. doi: 10.3390/ijerph18041670 (PMC7916252; doi:10.3390/ijerph18041670)
Supplement: Supplementary file 1 [file ijerph-18-01670-s001.pdf]

Table S1. Summary of findings of studies of Light Therapy in patients with Seasonal Affective Disorder, most recent first

Authors/ Date (most recent first)	Type of study/Use of placebo/ Light therapy (LT) monotherapy or as add on	Participants	Study protocol	Main outcomes
**Spies M. et al. (2018) [111]**	Case-control study<br>Placebo condition consisted of a non-biologically active light source at <400 lux<br>LT monotherapy	24 patients with SAD and 27 HC. All patients exhibited a Seasonal Problem Score ≥ 2 together with a Global Seasonality Score ≥ 10 on the Seasonal Pattern Assessment Questionnaire	In the two groups of the sample, changes in the baseline distribution of brain MAO-A in SAD compared to HC, after treatment with BLT and between seasons were studied using PET scans, which were performed in the fall / winter before and after 3 weeks of placebo-controlled BLT, as well as in spring / summer.<br>BLT treatment was administered over the course of 3 weeks daily, before noon, for 30 min, with an intensity of 10,000 lux.<br>Outcome measures: BDI and HDRS assessed in concomitance with the execution of PET scanning	Levels of cerebral MAO-A VT did not differ between patients and HC during the study.<br>Only bright light therapy showed a significant reduction in MAO-A VT
**Reeves G. M. et al. (2012) [112]**	Randomized controlled trial<br>Placebo condition was dim red light<br>LT monotherapy	79 participants with a diagnosis of current major depressive disorder with seasonal specifier and SIGH SAD of 21 or greater	This study has a randomized crossover design in which participants received in total 2 hours of treatment divided into 1 hour of bright light therapy (10,000 lux) and 1 hour of dim red light as a placebo.<br>Outcome measures: BDI-II and POMS-D measured at baseline and after each hour of light treatment using	By pooling the effects of light for the two sessions, there was a statistically significant reduction in self-reported depression scores in both BDI-II and POMS-D; in addition, a significant improvement, although modest, was documented after one single active light session
**Meesters Y, et al. (2011) [113]**	Randomized controlled trial<br>No placebo condition included<br>LT monotherapy	22 outpatients suffering from a MD with seasonal pattern	The study lasted two weeks and sessions were performed in 30 minutes during workdays. Patients were divided into two groups: one group received SLT at 10000 lux; the other group was treated with Blue-enriched white light at 750 lux. The patient sat at a distance of 20 cm from the light source.<br>Outcome measures: Weekly assessments and daily questionnaires at the beginning, during and at the end of the study were administered (including SIGH-SAD)	On all parameters, the effects of low-intensity blue-enriched white light in the treatment of SAD did not differ from the effects of exposure to standard bright-light treatment.<br>SIGH-SAD ratings were significantly reduced after treatment (SLT 65.2% and blue-enriched white light 76.4%)
**Flory R. et al. (2008) [114]**	Randomized controlled trial<br>Placebo conditions were low-density negative ions or dim red light<br>LT monotherapy or as add on to ongoing medication regimens	56 women affected by SAD selected during a 5 year-period	All patients were exposed to treatment for 12 consecutive days (30 min/day in the morning in January) and were split into 4 groups with different therapies:<br>-exposure to a 10 000-lux, 4100 K bright white light<br>-treatment with high-density (≥$2.0x10^6$ ions/cm^3) negative ions<br>-exposure to a 300-lux placebo dim red light<br>-treatment with placebo low-density (~$4.0x10^3$ ions/cm^3) negative ions<br>Outcome measures: SIGH-SAD-SR and BDI completed at baseline and again at the end of the 12th session of treatment, within 24 hours	With all treatments, significant scores improvements have been noted on both SIGH-SAD-SR and BDI, but, considering remission outcome criteria, treatment 1 has been the most effective and treatment 2 has achieved slightly better results than treatments 3 and 4
**Desan P. H. et al. (2007) [115]**	Randomized controlled trial<br>Inactivated negative ion generator worked as placebo condition	23 subjects with winter type SAD with a SIGH-SAD score of 20 or higher	At the randomization visit, subjects were assigned to either an active light treatment device (1,350 lux) or a placebo inactivated ion generator exposure condition. Each session lasted for 30 minutes a day upon awakening and prior to 8 a.m.<br>Outcome measures: Assessments were made during a baseline visit, a randomization visit and after 1, 2, 3 and 4 weeks of treatment, using SIGH-SAD. Participants were evaluated weekly during the 4 weeks of treatment	The proportions of participants in remission (with SIGH-SAD <9) were significantly higher and the individual percent scores at SIGH-SAD randomization were significantly lower with active bright light treatment than with control.

	LT monotherapy			A significant interaction between time and treatment, showing superiority of the bright light over the placebo condition, was also noticed
**Lam R. W. et al. (2006) [116]**	Randomized controlled trial, double-blind<br>Placebo condition was exposure to a 100-lux light and a placebo capsule<br>LT monotherapy	96 outpatients affected by Major Depressive Disorder characterized by seasonal pattern (during winter) with HDRS scores ≥ 23	This study lasted 8 weeks. Patients were split into two groups: the first group has been exposed to 10 000-lux by a fluorescent white-light box, in the morning, for 30 minutes/day and received a placebo capsule; the second one has been treated with 20 mg/day of fluoxetine and a 100-lux placebo light (with neutral density filters).<br>Outcomes measures: HDRS and SIGH-SAD; clinical response was defined as 50% or greater reduction from baseline in HDRS scores at the last visit	Both treatments showed similar results, with progressive improvements in patients' conditions.<br>The clinical response rate was 67% for both and the remission rates was 50% for group 1 and 54% for group 2.<br>Group 1 patients had more significant improvements after a week, but not at other assessments
**Terman M. et Terman S. J. (2006) [117]**	Randomized controlled trial<br>A deactivated ionizer was used as an inert placebo control<br>LT monotherapy	94 winter seasonal pattern Major Depressive Disorder patients and 5 Bipolar II patients were recruited in a 6-year period with at least 20 points on SIGH-SAD	The subjects went through a run-in phase (7-14 days) in which their sleep pattern was established with regular sleep schedules to be maintained throughout the study. Sample was then randomly divided into 5 subgroups:<br>-dawn simulation (0.0003–250 lux in the pattern of May 5 at 45°N latitude);<br>-a dawn light pulse (13 minutes, 250 lux, with an illuminant dose of 3.25×103 lux/minutes matched to the simulated dawn);<br>-post-awakening bright light (30 minutes, 10,000 lux);<br>-negative air ionization at high flow rate (93 minutes, 4.5×1014 ions/second);<br>-low-flow ionization (93 minutes, 1.7 × 1011 ions / second).<br>Outcome measures: SIGH-SAD assessed over the whole observation (3 weeks)	Post-treatment improvement results were bright light, 57.1%; dawn simulation, 49.5%; dawn pulse, 42.7%; high-density ions, 47.9%; and low-density ions, 22.7%
**Avery D. H. et al. (2001) [118]**	Randomized controlled trial<br>Placebo condition was exposure to a faint red light<br>LT monotherapy	95 outpatients with SAD and SIGH-SAD score ≥ 20.	After one week at baseline, the sample was randomly divided into three conditions: bright light therapy (10,000 lux for 30 min, 6:00 to 6:30), dawn simulation (1.5 hour sunrise signal 4:30 to 6:00 with a peak at 250 lux) and a placebo condition, a faint red light (1.5 hour sunrise signal from 4:30 to 6:00 with a peak of 0.5 lux).<br>Outcome measures: SIGH-SAD	Dawn simulation was achieved higher remission and response rates than the placebo condition and compared to bright light therapy.<br>Bright light did not appear to have any significant differences from placebo
**Wileman S. M. et al. (2001) [119]**	Randomized controlled trial<br>Exposure to dim red light served as placebo<br>LT monotherapy	57 participants with SAD with a score of minimum 15 in SIGH-SAD-SR	Subjects were randomly assigned to 4-week treatment of bright light (10000 lux) or dim red light. The administration protocol was: 30 min a day for the first week, 45 min a day for the second week and 1 h a day for the remaining weeks.<br>Outcome measures: SIGH-SAD-SR was compiled at baseline, weekly during treatment and 2 and 6 weeks after the end of the treatment	In both groups a 40% reduction of symptoms was observed, even if there were no differences in proportions of responders: 60 % met broad criteria for response and 31% met strict criteria.<br>No differences were found in treatment expectations
**Meesters Y. et al. (1999) [120]**	Randomized controlled trial<br>Placebo condition was no light exposure<br>LT monotherapy	38out patients with SAD and which suffered from regular annual depressions that already had experienced successful conventional light treatments in previous winters	Patients were assigned to three conditions:<br>- Condition 1: Exposure to bright white visor light<br>- Condition 2: Exposure to infrared light by means of a light visor equipped with a Kodak Wratten filter<br>- Condition 3: No light exposure; no light visor provided.<br>Outcome measures: BDI and SIGH-SAD-SR assessed weekly	Infrared light seemed to be just as effective as bright white light.<br>Both kind of treatments showed to be more effective than the control condition with no light exposure
**Eastman C. I. et al. (1998) [121]**	Placebo-controlled trial	96 outpatients with SAD and 21 or more on SIGH-SAD	Patients were randomly allocated to 1 of 3 groups for a treatment of 4 weeks, each 1.5 hours per day, using bright light (6000 lux) generated by light boxes, except for	No differences among the 3 groups in mean depression scores or expectation ratings after 4 weeks of treatment were found in this study.

	Placebo condition was exposure to false negative ion generators<br>LT monotherapy		the placebo-group: the first group was exposed to light in the morning; the second one received light in the evening; the placebo condition happened in the morning.<br>Outcome measures: SIGH-SAD assessed weekly	Morning light seemed to produce more complete or near complete remissions than placebo after 3 weeks of treatment.<br>Considering SIGH-SAD score <50% of baseline and a result ≤8, after 4 weeks of treatment 61% of the patients showed to respond to morning light, 50% to evening light and 32% to placebo
**Lewy A. J. et al. (1998) [122]**	Case-control study<br>No placebo condition included<br>LT monotherapy	51 outpatients with winter-type SAD and 49 healthy controls with a score 20 or higher on the SIGH-SAD	Subjects were studied for 6 weeks after a period of light/dark and sleep/wake adaptation that lasted 1 week. Patients and HC underwent bright light exposure (2500 lux) for 2 weeks for a 2-hour session either in the morning or in the evening and they were crossed over to the other light schedule after one week of withdrawal from any light treatment.<br>Outcome measures: SIGH-SAD scale was administered at baseline, during withdrawal weeks and at the end of the observation; dim-light melatonin onsets were obtained 7 times during the study to assess circadian phase position	Dim-light melatonin onsets were generally postponed in patients compared with HC.<br>The morning light phase anticipated melatonin onset in dim light and appeared to be more antidepressant than evening light, which delayed it
**Ruhrmann S. et al. (1998) [123]**	Randomized controlled trial<br>Placebo condition was exposure to dim light and taking an inert capsule<br>LT monotherapy	35 outpatients suffering from SAD who had a total score of at least 16 on the HDRS	The observation was divided into 2 phases: in phase I patients received one week of placebo (dim light and capsule) in order to test response to placebo<br>During phase II, that lasted 5 weeks, placebo non-responders were randomly assigned to 2 groups: the first group took fluoxetine (20 mg per day) and a placebo light condition (2 h per day); the other group received bright light (3000 lux, 2 hours a day) and a placebo pill.<br>Outcome measures: HDRS and Hypomania scale monitored weekly	70% of patients treated with bright light and 65% of those treated with fluoxetine were responders and the remission rate in the bright light group tended to be higher (bright light 50%, fluoxetine 25%).<br>Light therapy improved HDRS scores significantly faster than fluoxetine.<br>Receiving light treatment in the morning caused a significantly faster improvement, but at the end of the treatment the timing of light administration during the day did not seem to be decisive
**Terman M. et al. (1998) [124]**	Randomized controlled trial<br>Placebo condition involved exposure to negative air ionization<br>LT monotherapy	145 subjects who met SAD criteria and criteria for current major depressive episode	The observation lasted a total of 6 years.<br>In this study there were 5 groups of subjects treated with bright light (10000 lux, 30min/d) and 1 group of controls exposed to negative air ionization, all for 2 consecutive treatment periods, each 10 to 14 days.<br>The groups treated with bright light differed according to the sequence of exposure: morning-evening, evening-morning, morning-morning and evening-evening.<br>The ion density sequences could have been high, high-and-low or low and were performed for 30 min/d in the morning.<br>Outcome measures: SIGH-SAD along the treatment period	Patients exhibited a significantly greater response to morning light than to evening light, regardless of the sequence used during treatment.<br>The obtained results seem to indicate that the low-density ion response is lower than all the other groups, with no other group differences.<br>The only effect of the sequence was that the evening light response was reduced if the morning light treatment was done earlier
**Michalon M. et al. (1997) [125]**	Case-control study.<br>Placebo condition included exposure to dim red light.<br>LT monotherapy	29 SAD outpatients with a current depressive episode and 29 HC. Patients had at least a score of 20 in SIGH-SAD at the assessment visit	This study examined the performance of SAD patients on a wide range of cognitive variables before and after 2 weeks of light treatment with either white (2500 lux) or placebo red light, as well as in the summer period (only 13 patients); patients were randomly assigned to one of the 2 treatment groups.<br>The treatment sessions were held between 06:00 and 08:00 every day for 2 consecutive weeks between October and February.<br>The performance of the SAD subjects was then compared with a group of 29 HC standardized for age and education, who did not receive light therapy.<br>Outcome measure was SIGH-SAD	Treatment with bright light showed good results on measures of psychiatric symptoms, but no change in reports of cognitive failures.<br>The results on visual memory and constructive deficits on treatment with white light or the presumed red-light placebo were non-specific

**Partonen T. et Lönnqvist J. (1996) [126]**	Randomized controlled trial<br>No placebo condition<br>LT monotherapy	12 female outpatients with winter SAD and at least a score of 16 in SIGH-SAD-SR and a score of 10 in HDRS	Patients were divided into two groups: the first group started treatment with bright light therapy before onset of symptoms and the second group was treated after onset of symptoms. For each group, bright light (3300 lux) was administered at first for 1 hour daily for 1 week; after the first week, patients were given the option of continuing with a schedule of their choice (morning, evening, or combined exposure) for as long a period as they wished until a pre-established deadline.<br>Outcome measures: SIGH-SAD-SR and HDRS	There were no significant changes in the SIGH-SAD-SR subscale scores. The HDRS subscale scores, instead, showed that clinical remission was significantly more frequent in the first subgroup of patients and suggested that bright light therapy given in advance of the emerging symptoms was effective in preventing a new depressive episode
**Levitt A. J. et al. (1994) [127]**	Randomized controlled trial<br>Placebo condition was exposure to dim light<br>LT monotherapy or as add on to ongoing medication	43 SAD outpatients with a score greater than 12 on HDRS greater than 10 on SIGH-SAD	Sample was randomly divided in 2 groups to receive 2 weeks of treatment with either bright- (4106 lux) or dim-light, using red light-emitting diode light sources; each session was conducted 30 min each morning before 9 am.<br>Outcome measures: SIGH-SAD (performed at baseline, repeated at weeks 1 and 2 of treatment and after 1 week off light therapy), CGI-I (at the end of 2 weeks of light therapy. Clinical response to treatment was determined by a decrease in HDRS scores from baseline at the end of observation	Assuming a clinical response value of a 50% reduction in HDRS score at a post-treatment score <8, no significant difference was found in the response rate between patients who received bright light (67%) compared to patients who received dim light (68%)
**Martinez B. et al. (1994) [128]**	Randomized controlled trial, single-blind<br>Placebo condition was exposure to dim light<br>LT as add on to hypericum extract	20 SAD outpatients with a total of HDRS score of at least 16 after a one-week washout phase	Patients were randomized to 2 groups: the first received additional phototherapy with a bright white light (3000 lux) and the latter group was given dim light. Each session of light therapy lasted for 2 hours daily for 4 weeks in total.<br>All groups received treatment with three 300 mg tablets of hypericum extract per day during the whole observation period.<br>Outcome measures: HDRS, HS, ES, POMS, the von Zerssen self-assessment scale, the von Zerssen depression scale and the Visual Analogue scales	Relevant HDRS scores' reduction was found in both groups, with no significant differences
**Eastman C. I. et al. (1992) [129]**	Randomized controlled trial<br>Exposure to deactivated negative ion generator served as placebo condition<br>LT monotherapy	32 SAD outpatients with a current depressive episode and a score>21 in HDRS	The study used a 5-week counterbalanced crossover design with one week of baseline, two consecutive weeks of light treatment (1 h a day of light treatment with 7000 lux) and two consecutive weeks of placebo treatment (1 h a day of placebo treatment with deactivated negative ion generator), both administered in the morning.<br>Outcome measures: daily and weekly questionnaires (Daily Sleep Log; Daily Ion and Light Log; BDI; SIGH-SAD)	Both protocols led to a significant reduction in depression ratings, but no relevant difference was found between the clinical response to light and placebo
**Magnusson A. et Kristbjarnarson H. (1991) [130]**	Placebo-controlled crossover trial<br>Placebo condition was exposure to red light<br>LT monotherapy	10 outpatients with a history of at least one major depressive episode, regularly occurring fall-winter depression alternating with remission during spring and summer	For 8 days-period patients were divided into 2 groups: the first received 40-min exposure to 10,000 lux white light and the latter got an exposure to 400 lux red light, which served as placebo. The patient sat at a distance of 35 cm from the light source. After a wash-out period partecipants was crossed over to the other treatment condition.<br>Outcome measures: HDRS, SIGH-SAD and BDI at the beginning and at the end of each treatment period	The results have shown greater improvement with 10,000 lux treatment than with placebo.<br>The 10,000-lux therapy improved the SIGH-SAD score by an average of 16.1 while the average improvement on placebo treatment was 5.0

SAD=Seasonal Affective Disorder; HC=Healthy Controls; MAO A (VT)= MonoAmyno-Oxydase A (Total distribution Volume); BLT=Bright Light Therapy; PET=Positron Emission Tomography; BDI/BDI-II=Beck Inventory Scale; HDRS=Hamilton Depression Rating Scale; POMS-D=Profile Of Mood States-Depression-dejection subscale; MD=Major Depression; SLT=Standard Light Therapy; SIGH-SAD (-SR)= Structured Interview Guide for the Hamilton Depression Rating Scale-Seasonal Affective Disorder Version (-Self Rating Version); IR=InfraRed Light; CON=No Light exposure; CGI-I=Clinical Global Impression of Improvement; HS=Hypomania Scale; ES=Expectation Scale; Bf-S= von Zerssen self-assessment scale; D-S= von Zerssen depression scale.

Table S2. Summary of findings of studied using Light Therapy in patients with Major Depressive Disorders (Unipolar depression and Bipolar Disorder) most recent first

Authors/ Date (most recent first)	Type of study/Use of placebo/ Light therapy (LT) monotherapy or as add on	Participants	Study protocol	Main outcomes
**Chan et al. (2020) [142]**	Randomised controlled trial<br>Placebo condition was exposure to dim red light<br>LT monotherapy	93 outpatients with unipolar non-seasonal depression with evening chronotype and scoring at least 14 on SIGH-SAD and scoring 41 or less in the MEQ	5-week treatment, lasting for 30 min/day and 5 months follow up. Bright light therapy: 10000 lux, dim red light: 50 lux.<br>Light therapy was started within 1 hour of the habitual wake time<br>Outcome measures: participants were clinicaly assessed at baseline, weekly during active intervention, at 1-week post-treatment, 1-month post-treatment, 2-month post-treatment and 5-month post-treatment	Bright light therapy with gradual advance protocol resulted in quicker and a higher rate of remission of depression in patients with non-seasonal unipolar depression and evening-chronotype. No significant differences between the two groups for anxiety symptoms, insomnia severity, suicidal ideation and fatigue
**Danilenko et al. (2019) [143]**	Randomised controlled trial.<br>No placebo condition<br>LT as add on to partial sleep deprivation	35 inpatients with moderately severe non seasonal depressive disorders and score at least 12 to HDRS and score at least 16 to BDI	6-day protocol: 3 consecutive 2-day cycles comprising partial sleep deprivation (04.00-08.00) in a light therapy room (blue-enhanced white light increased hourly from 600→1300→2200→2800 lux) alternating with recovery nights with morning light treatment from 7:00 to 8:00. Randomized to wear glasses with no filter (clear, N=19) or filtering blue wavelength (orange appearance, light intensity diminution by ~70%, N=16) during the treatment<br>Outcome measures: HDRS, BDI, and VAS	Depression levels significantly declined; no difference between white and orange lights. Some superiority of white light emerged with respect to response rate (mood VAS), immediate effect during the 4-h treatment sessions (energy VAS), and expected treatment outcomes
**Kupeli et al. (2018) [144]**	Randomized controlled trial, single-blind.<br>Placebo condition was exposure to dim light<br>LT as add on to ongoing drug therapy	32 outpatients with bipolar disorders (16 for each group); HDRS scores ≥ 17	Patients were randomly assigned to bright light therapy (10000 lux) or dim light (<500 lux). 2 week of treatment, 30 min each morning (between 08.00-10.00). No change in treatment regimens (except lorazepam as an add-on treatment).<br>Outcome measures: HDRS and MADRS	Statistically significant reduction in depression score for the bright light group (81% good treatment response, 44% remission of criteria)
**Sikkens et al. (2018) [145]**	Cohort study.<br>No placebo condition included<br>LT as add on to TSD and ongoing drug therapy	Twenty-six depressed patients with unipolar or bipolar depression	14-day program consisting of three nights of TSD, alternated with recovery nights and ten sessions of LT on working days between 06:30 and 07:00, using bright white light (10.000 lux). On the days after the TSD nights, patients were allowed to go to bed at 18:00. Patients continued the use of antidepressants (in MDD) or lithium (in BD) or started the medication 1 week before the start of the treatment.<br>Outcome measures: IDS-C scores were determined before chronotherapy and at week 1, 2, and 4	Clinically relevant reduction of severity of depression was observed after the first week of treatment and were maintained for at least a 4-week period.<br>Patients with psychiatric comorbidity showed a lower reduction in IDS-C scores but still experienced a significant drop in depression severity
**Sit et al. (2018) [97]**	Randomized double-blind placebo-controlled trial<br>LT as add on to ongoing drug therapy	46 depressed adults with bipolar I or II disorder with stable medication, a score ≥ 20 on the SIGH-ADS and no hypomania or mixed symptoms	6-week of treatment with adjunctive light therapy at midday. Patients were randomly assigned to treatment either to a 7,000-lux, 4,000-K, broad-spectrum white fluorescent or a 50-lux red light unit. Patients began with 15-minute sessions of light therapy between 12:00 p.m. and 2:30 p.m increased by 15 minutes' intervals to attain a target dose of 60 minutes per daily session by week 4 or until remission. Patients were stratified by antidepressant use with a block design.<br>Outcome measures: SIGH-ADS, the Mania Rating Scale, and the Pittsburgh Sleep Quality Index. Remission was defined as having a SIGH-ADS score of 8 or less	After 6 weeks of bright light therapy, 68.2% experienced remission, and patients reported low levels of depression, significantly better global functioning, and no mood polarity switch. With dim red light, only 22.2% remitted. No mood polarity switches were observed

**Zhou et al. (2018) [105]**	Single blind, randomized controlled trial. Placebo condition was exposure to dim red light LT as add on to ongoing drug therapy	74 participants were randomized and 63 participants completed the study (33 bright light, 30 dim red light) with score of 17 or more to HDRS at baseline	Bright light (5000 lux at 100 cm distance) and dim red light (less than 100 lux) was administered every morning for 1 h between 6:30 a.m. to 9:00 a.m Outcome measures: HDRS, CGI , SERS , QIDS-SR16	Bright light therapy showed a greater ameliorative effect on bipolar depression than the placebo condition. No participants experienced symptoms of hypomania. No serious adverse events were reported
**Chojnacka et al. (2016) [146]**	Randomized controlled trial. Placebo condition was exposure to negative ion generator LT as add on to ongoing drug therapy	95 patients (50 bipolar disorders and 45 unipolar disorders) with current major depressive episode for at least 6 weeks, on stable treatment for at least 4 weeks prior to enrolment with insufficient treatment effects (CGI ≥3)	52 patients were randomized to the bright light therapy and 43 to the placebo group. 2 weeks of treatment, no change in medication. 30 min of light therapy at 10,000 lux half an hour after waking between 8 and 9 a.m. Outcome measures: HDRS, MADRS, BDI, CGI, PGI	Subjects treated with light therapy did not significantly differ in terms of improvement in HDRS scores compared to placebo. Higher response and remission rates in light group. Light therapy was more effective in drug-resistant patients. No significant differences between unipolar and bipolar disorders
**Lam et al. (2016) [147]**	Randomised controlled trial, double-blind. Placebo condition was exposure to inactive negative ion generator + placebo pill LT monotherapy or as add on to fluoxetine	122 outpatient with Major Depressive Disorders of at least moderate severity with ≥ 20 to HDRS	8-weeks treatment. Patients randomly assigned to:<br>-light monotherapy (active 10 000-lux fluorescent white light box for 30 min/day in the early morning + placebo pill) (n=32)<br>-antidepressant monotherapy (inactive negative ion generator for 30 min/day + fluoxetine hydrochloride, 20mg/d) (n=31)<br>-combination light and antidepressant (n=29)<br>-placebo (inactive negative ion generator + placebo pill) (n=30).<br>Outcome measure: MADRS, CGI, QIDS-SR	Bright light treatment, both as monotherapy and in combination with fluoxetine, had significant benefits compared with a sham-placebo condition. The combination treatment had the most consistent effects
**Suzuki et al. (2016) [148]**	Cohort study. No placebo condition included LT as add on to TSD and lithium	149 inpatients with a major depressive episode without psychotic features in the course of bipolar disorder type I, with a HDRS score ≥ 18	Patients were treated with the combination of repeated sleep deprivation and bright light therapy. 3 consecutive TSD cycles (day 0,2,4 totally sleep deprived from 07.00 until 19.00 of the following day; sleep the night 19.00-08.00 of day 1,3,5) + 30 min to 10000 lux bright white light at 03.00 during the TSD night and in the morning after recovery sleep (half an hour after awakening, between 08.00-09.00). Light therapy in the morning was continued for 2 weeks. Combination therapy with lithium, no other antidepressant.<br>Outcome measures: BDI (self-rated) and HDRS (observer-rated). BDI-HDRS discrepancy score (discrepancy between subjective and objective severity of depression) at baseline was calculated	Response rate of the low discrepancy group was significantly higher than that of the high discrepancy group (80.2%vs.48.5%). High BDI-HDRS discrepancy predicted negative response to treatment
**Camardese et al. (2015) [149]**	Open-label study. No placebo condition included	31 depressed outpatients (16 unipolar and bipolar); 25 patients completed the treatment and 5 weeks of follow up	Daily treatment between 5.45 am and 8.15 am; exposure of 30 minutes at 10,000 lux for 3 weeks. Schedule was defined following MEQ scores. After the first week of treatment, partial or no responders were instructed to increase the exposure to 45 minutes/day for the next 2 weeks.	Bright light therapy seemed to influence the course of the depressive episode, with a statistically significant reduction in HDRS scores since week 1 of therapy. No (hypo)manic switch. After follow-up 9 patients (36%,

	LT as add on to ongoing drug therapy		Outcome measures: HDRS, Snaith-Hamilton Pleasure scale (anhedonia), Depression Retardation Rating scale (psychomotor retardation)	eight unipolar and one bipolar) still showed a treatment response
**Benedetti et al. (2014) [150]**	Cohort study. No placebo condition included LT as add on to TSD and lithium	141 inpatients with a major depressive episode without psychotic features in the course of bipolar disorder, with a HDRS score ≥ 18. 32 had a positive history of suicidality	3 consecutive TSD cycles (day 0,2,4 totally sleep deprived from 07.00 until 19.00 of the following day; sleep the night 19.00-08.00 of day 1,3,5) + 30 min to 10000 lux bright white light at 03.00 a.m. during the TSD night and in the morning after recovery sleep (half an hour after awakening, between 08.00-09.00). Light therapy in the morning was continued for 2 weeks. Combination therapy with lithium, no other antidepressant. Patients were followed for 1 month after treatment. Outcome measures: BDI, HDRS	70% response rate after 1 week, 55.3% of patients with sustained response. 1.4% manic switch. Immediate decrease in suicide risks cores after first cycle of total sleep deprivation and light therapy. Synergic effect of lithium and light therapy with sleep deprivation
**Dauphinais et al. (2012) [151]**	Randomized controlled trial. Placebo condition was exposure to negative ion generator LT as add on to ongoing therapy	44 adults with bipolar disorder during a depressive episode with a score of 20 or more to SIGH-SAD	Subjects were randomized to one of three treatment groups (bright light therapy, low-density or high-density negative ion generator). Treatment and follow up lasted 8 weeks. Treatment duration began at 7.5 min/day, increased at 7.5-min increments, to a maximum exposure of 45 min/day. Light therapy was administered at 10,000 and 7000 lux. Outcome measures: SIGH-ADS, YMRS and SAFTEE (for adverse effects)	Results showed no statistically significant differences between groups in any outcome measures at study end; adverse events, including switches into hypomania, were rare
**Benedetti et al. (2007) [152]**	Cohort study. No placebo condition included LT as add on to TSD and ongoing drug therapy	39 inpatients affected by Type I Bipolar Disorder (current depressive episode without psychotic features). 13 had a medical history of drug resistance	1 week with repeated TSD (36 h) combined with morning light therapy (exposure for 30 min to a 400 lux green light: at 03:00 h during the TSD night and in the morning after recovery sleep, half an hour after awaking). Patients were then allowed to sleep during the night of days 2, 4, and 6. TSD was carried out in a room with roughly 80 lux ambient light. 14 patients were on ongoing lithium medication, 9 SSRI, 3 venlafaxine Outcome measures: HDRS (on days 1, 2, 3, and 7); VAS (self-administered 3 times per day), wrist actigraphy	2/3 of the patients responded to treatment (50% reduction in HDRS score). The antidepressant response to repeated TSD and light therapy treatment was paralleled by changes in the activity-rest rhythm and sleep
**Martiny et al. (2006) [153]**	Randomised controlled trial, double-blind. Placebo condition was exposure to dim red light LT as add on to sertraline	92 patients with major non-seasonal depression and a score >13 on HDRS. 43 patients treated with bright light	5 weeks of treatment with sertraline combined with bright-light therapy (1 h in the morning 10000 lux) or sertraline combined with dim-light therapy (100 lux for 30 min in the morning). During the first 5 weeks fixed dose of Sertraline 50 mg. At the beginning of the follow-up (4 weeks) the sertraline treatment continued, while the light therapy stopped. Outcome measures: HDRS, SIGH-SAD	Depression scores decreased in both groups during the 9 weeks of experiment. At week 5 better results for light group, the improvement disappeared gradually in the 4-week follow-up period, resulting in similar end-point scores
**Goel et al. (2005) [154]**	Randomized controlled trial Placebo condition was exposure to low density negative ions LT monotherapy	32 patients. Major depressive disorders, single episode, duration ≥ 2 years	Bright light (10 000 lux, n=10), high-density ($4{\cdot}5 \times 10^{14}$ ions/s flow rate, n=12) or low-density ($1{\cdot}7 \times 10^{11}$ ions/s, n=10, placebo control) negative ions. Home treatment sessions, for 1 h within 10 min of waking for 5 weeks. Evening saliva samples obtained before and after treatment for ascertainment of circadian melatonin rhythm phase. Outcome measures: SIGH-SAD	SIGH-SAD score improvements for both experimental condition (bright light and negative air ions) Light and negative ions are candidate adjuncts to drugs and psychotherapy for chronic depression
**McEnany et al. (2005) [155]**	Randomized controlled trial. Placebo condition	29 pre-menopausal and	Patients were randomized either to light therapy with 2500 lux intensity via a visor, in the morning during the first waking hour or to a placebo delivered wearing a pair	Significant changes in depression and energy levels, and wake time reduction during the first third of the sleep

	was wearing a pair of circadian adaptation glasses<br>LT monotherapy	post-menopausal women with nonseasonal, unipolar depression	of “circadian adaptation glasses” designed to filter out light one hour before bedtime. Treatment duration for both arms 28 days.<br>Outcome measures: BDI, VAS, body temperature, sleep stages by EEG and electrooculogram	period, in the light therapy group but not in the placebo group. There was no significant phase shift
**Martiny et al. (2004) [156]**	Randomised controlled trial, double-blind.<br>Placebo condition was exposure to dim red light<br>LT as add on to sertraline	102 outpatients with a diagnosis of non-seasonal major depression and score of more than 13 on HDRS	5 weeks of daily treatment with sertraline combined with bright-light therapy (1 h in the morning 10000 lux) or sertraline combined with dim-light therapy (50 lux for 30 min in the morning).<br>Sertraline in a fixed dose of 50 mg daily<br>Outcome measures: HDRS, MDI, PGWB, SCL-90R	Reduction in depression scores was larger in the bright light group
**Benedetti et al. (2003) [157]**	Randomized controlled trial<br>Placebo condition was exposure to deactivated negative ion generator<br>LT as add on to citalopram	21 inpatients with major depressive disorder, 9 inpatients with bipolar disorder. None of the patients had a seasonal pattern	All patients were treated with citalopram per os, started at 10 mg/day at day 1, then rapidly titrated to 40 mg/day at day 4; same dose continued until week 4. Randomized in a 3:2 manner to receive 30 min of 400 lux green light treatment (spectrum ranging 485-515 nm) in the morning or placebo during the first 2 weeks of the drug treatment. Timing was individually defined and calculated with MEQ; placebo exposure was 1.5 h after the optimal timing. Study duration 4 week.<br>3 outcome measures: HDRS, SDS, VAS (self-rated during the first week 3 times/day)	Morning light therapy was superior to placebo in augmenting the antidepressant effect of citalopram. The effect began the first day of treatment (shown by self-ratings of perceived mood) and continued throughout the 4 weeks: HDRS scores decreased to 31.2% of baseline levels in light therapy patients and to 58% in placebo patients
**Loving et al. (2002) [158]**	Randomized controlled trial.<br>Placebo condition was exposure to dim red light<br>LT monotherapy	13 outpatients with major depressive disorders, with no seasonal traits	Half night of home wake treatment, followed by 1 week of light treatment; 30 min between 6-9 a.m. Randomly assigned to 10000 lux bright white or dim red light (100 lux)<br>Outcome measures: SIGH-SAD	Light therapy group improved of 27% on SIGH-SAD in 1 week, no improvement in the placebo group
**Prasko et al. (2002) [159]**	Randomized controlled trial, double blind.<br>Placebo condition was exposure to dim red light)<br>LT monotherapy or as add on to imipramine	34 inpatients with major depressive disorders recurrent type. HDRS ≥ 20	4-day washout period with baseline assessment, then 3 weeks of treatment.<br>Group A: bright light therapy (5000 lux from 6–8 a.m.) and imipramine 150 mg/day.<br>Group B: bright light therapy (5000 lux from 6–8 a.m.) and imipramine-like placebo.<br>Group C: dim red light (500 lux from 6–8 a.m.) and imipramine 150 mg/day.<br>Outcome measures: HDRS, CGI, MADRS and BDI (weekly)	The efficacy of bright light therapy alone was comparable with that of the imipramine treatment, and better than the combination of bright light and imipramine (not statistically significant)
**Beauchemin et Hays (1997) [160]**	Randomized controlled trial, single-blind.<br>No placebo condition<br>LT as add on to ongoing drug therapy	22 inpatients were recruited: 19 completed the trial. Of these, 10 subjects fulfilled the criteria for bipolar disorder	Subjects were randomly assigned to receive high (10000 lux) or low levels (2500 lux) of light therapy. The treatment duration was 30 min/day for 1 week and was administered between 07.30 and 09.30.<br>Outcome measures: daily clinical assessment from the researcher and by POMS-B (self-rated scale) assessed on day 1 and day 7	Patient with both unipolar and bipolar depression responded when light therapy was used as an adjunct to pharmacotherapy. Improvement was related to the intensity of illumination. 3 patients (receiving high levels of light) dropped out of the experiment because their mood had improved markedly

**Yamada et al. (1995) [161]**	Case-control study.<br>Placebo condition was exposure to dim light<br>LT monotherapy	27 inpatients with major depressive disorders (17 major depression and 10 bipolar depression) + 16 healthy controls. None of the patients had a seasonal pattern	Randomly assigned to morning (06.00-08.00) or evening (18.00-20.00) treatment and to bright (2500 lux) or dim (500 lux) light therapy of seven-day duration. Bright light: 12 x 20 w cool-white light fluorescent full-spectrum (including ultraviolet) light tubes. Dim light was induced by the same apparatus, mounted behind a yellow transparent film.<br>Outcome measures: HRSD was used for rating patient symptoms by a psychiatrist who was blind to the types of exposure to light	Bright light significantly reduced the severity of depression; the therapeutic effect was not observed after dim light. Both morning and evening exposure to bright light gave a therapeutic effect
**Kripke et al. (1994) [162]**	Randomised controlled trial, double blind.<br>Placebo condition was exposure to dim red light<br>LT monotherapy	51 inpatients with nonseasonal major depressive disorders or depressed episodes of bipolar disorder and Scores of at least 15 on HDRS and BDI	25 patients treated with light for 1 week (2000-3000 lux); 26 patients treated with dim red light. 32 were treated from 8:00-11:00 PM and 7 were treated from 7:00-10:00 PM (to accommodate patient's habits)<br>Outcome measures: HDRS, BDI	Patients treated with bright white light showed a reduction in depression during treatment. Partial relapse appeared within 2 days. Two bright-light-treated patients became mildly hypomanic, but side effects were mild
**Deltito et al. (1991) [135]**	Case-control study.<br>Placebo condition was exposure to 400 lux light<br>LT monotherapy	17 out-patients with non-SAD depressive disorders with ≥ SIGH-SAD 18 score. Subjects included 6 patients with major depressive episodes and 6 bipolar II patients	All patients were treated with either 400 or 2500 lux phototherapy for 2 h (within 1 h of their normal waking time) on seven consecutive days.<br>Outcome measures: SIGH-SAD, CGI, and the Anxiety and Depressive Factors of the SCL-90	Outcome measures showed greater improvement in the bipolar vs. the unipolar spectrum patients regardless of the intensity of the light. All patients showing response were noted to have maintained their response at a 3-month follow-up

HDRS= Hamilton Rating Scale for Depression; TSD= Total Sleep Deprivation; SDS= Zung Self-Rating Depression Scale; VAS= Visual Analogue Scale; POMS-B=Profile of Mood States - Bipolar Form; BDI=Beck Depression Inventory; IDS-C= Inventory of Depressive Symptoms C; CGI= Global Assessment Scale; SIGH-SAD = Structured Interview Guide for the Hamilton Depression Rating Scale - Seasonal Affective Disorder Version; SCL-90= Symptom Check List 90; SIGH-ADS= Structured Interview Guide for the Hamilton Depression Rating Scale with Atypical Depression Supplement; YMRS= Young Mania Rating Scale; SAFTEE= Systematic Assessment for Treatment Emergent effects; SERS= Side Effects Rating Scale of Asberg; QIDS-SR16= 16-item Quick Inventory of Depressive Symptomatology, Self-report; MADRS= Montgomery-Asberg Depression Rating Scale; MEQ= Morningness–Eveningness Questionnaire; PGI= Patient Global Impression; MDI= Major Depression Inventory; PGWB= The Psychological General Well-Being Scale

Reference

97. Sit, D.K.; McGowan, J.; Wiltrout, C.; Diler, R.S.; Dills, J.; Luther, J.; Yang, A.; Ciolino, J.D.; Seltman, H.; Wisniewski, S.R.; et al. Adjunctive Bright Light Therapy for Bipolar Depression: A Randomized Double-Blind Placebo-Controlled Trial. *AJP* **2018**, *175*, 131–139, doi:10.1176/appi.ajp.2017.16101200.
105. Zhou, T.; Dang, W.; Ma, Y.; Hu, C.; Wang, N.; Zhang, G.; Wang, G.; Shi, C.; Zhang, H.; Guo, B.; et al. Clinical Efficacy, Onset Time and Safety of Bright Light Therapy in Acute Bipolar Depression as an Adjunctive Therapy: A Randomized Controlled Trial. *J. Affect. Disord.* **2018**, *227*, 90–96, doi:10.1016/j.jad.2017.09.038.
111. Spies, M.; James, G.M.; Vraka, C.; Philippe, C.; Hienert, M.; Gryglewski, G.; Komorowski, A.; Kautzky, A.; Silberbauer, L.; Pichler, V.; et al. Brain Monoamine Oxidase A in Seasonal Affective Disorder and Treatment with Bright Light Therapy. *Transl. Psychiatry* **2018**, *8*, 198, doi:10.1038/s41398-018-0227-2.
112. Reeves, G.M.; Nijjar, G.V.; Langenberg, P.; Johnson, M.A.; Khabazghazvini, B.; Sleemi, A.; Vaswani, D.; Lapidus, M.; Manalai, P.; Tariq, M.; et al. Improvement in Depression Scores After 1 Hour of Light Therapy Treatment in Patients With Seasonal Affective Disorder. *J. Nerv. Ment. Dis.* **2012**, *200*, 51–55, doi:10.1097/NMD.0b013e31823e56ca.
113. Meesters, Y.; Dekker, V.; Schlangen, L.J.M.; Bos, E.H.; Ruiter, M.J. Low-Intensity Blue-Enriched White Light (750 Lux) and Standard Bright Light (10,000 Lux) Are Equally Effective in Treating SAD. A Randomized Controlled Study. *BMC Psychiatry* **2011**, *11*, 17, doi:10.1186/1471-244X-11-17.
114. Flory, R.; Ametepe, J.; Bowers, B. A Randomized, Placebo-Controlled Trial of Bright Light and High-Density Negative Air Ions for Treatment of Seasonal Affective Disorder. *Psychiatry Res.* **2010**, *177*, 101–108, doi:10.1016/j.psychres.2008.08.011.
115. Desan, P.H.; Weinstein, A.J.; Michalak, E.E.; Tam, E.M.; Meesters, Y.; Ruiter, M.J.; Horn, E.; Telner, J.; Iskandar, H.; Boivin, D.B.; et al. A Controlled Trial of the Litebook Light-Emitting Diode (LED) Light Therapy Device for Treatment of Seasonal Affective Disorder (SAD). *BMC Psychiatry* **2007**, *7*, 38, doi:10.1186/1471-244X-7-38.
116. Lam, R.W.; Levitt, A.J.; Levitan, R.D.; Enns, M.W.; Morehouse, R.; Michalak, E.E.; Tam, E.M. The Can-SAD Study: A Randomized Controlled Trial of the Effectiveness of Light Therapy and Fluoxetine in Patients with Winter Seasonal Affective Disorder. *Am. J. Psychiatry* **2006**, *163*, 805–812, doi:10.1176/ajp.2006.163.5.805.
117. Terman, M.; Terman, J.S. Controlled Trial of Naturalistic Dawn Simulation and Negative Air Ionization for Seasonal Affective Disorder. *Am. J. Psychiatry* **2006**, *163*, 2126–2133, doi:10.1176/ajp.2006.163.12.2126.
118. Avery, D.H.; Eder, D.N.; Bolte, M.A.; Hellekson, C.J.; Dunner, D.L.; Vitiello, M.V.; Prinz, P.N. Dawn Simulation and Bright Light in the Treatment of SAD: A Controlled Study. *Biol. Psychiatry* **2001**, *50*, 205–216, doi:10.1016/S0006-3223(01)01200-8.
119. Wileman, S.M.; Eagles, J.M.; Andrew, J.E.; Howie, F.L.; Cameron, I.M.; McCormack, K.; Naji, S.A. Light Therapy for Seasonal Affective Disorder in Primary Care: Randomised Controlled Trial. *Br. J. Psychiatry* **2001**, *178*, 311–316, doi:10.1192/bjp.178.4.311.
120. Meesters, Y.; Beersma, D.G.; Bouhuys, A.L.; van den Hoofdakker, R.H. Prophylactic Treatment of Seasonal Affective Disorder (SAD) by Using Light Visors: Bright White or Infrared Light? *Biol. Psychiatry* **1999**, *46*, 239–246, doi:10.1016/s0006-3223(98)00252-2.
121. Eastman, C.I.; Young, M.A.; Fogg, L.F.; Liu, L.; Meaden, P.M. Bright Light Treatment of Winter Depression: A Placebo-Controlled Trial. *Arch. Gen. Psychiatry* **1998**, *55*, 883–889, doi:10.1001/archpsyc.55.10.883.
122. Lewy, A.J.; Bauer, V.K.; Cutler, N.L.; Sack, R.L.; Ahmed, S.; Thomas, K.H.; Blood, M.L.; Jackson, J.M. Morning vs Evening Light Treatment of Patients with Winter Depression. *Arch. Gen. Psychiatry* **1998**, *55*, 890–896, doi:10.1001/archpsyc.55.10.890.
123. Ruhrmann, S.; Kasper, S.; Hawellek, B.; Martinez, B.; Höflich, G.; Nickelsen, T.; Möller, H.J. Effects of Fluoxetine versus Bright Light in the Treatment of Seasonal Affective Disorder. *Psychol. Med.* **1998**, *28*, 923–933, doi:10.1017/s0033291798006813.
124. Terman, M.; Terman, J.; Ross, D. A Controlled Trial of Timed Bright Light and Negative Air Ionization for Treatment of Winter Depression. *Arch. Gen. Psychiatry* **1998**, *55*, 875–892.
125. Michalon, M.; Eskes, G.A.; Mate-Kole, C.C. Effects of Light Therapy on Neuropsychological Function and Mood in Seasonal Affective Disorder. *J. Psychiatry Neurosci.* **1997**, *22*, 19–28.
126. Partonen, T.; Lönnqvist, J. Prevention of Winter Seasonal Affective Disorder by Bright-Light Treatment. *Psychol. Med.* **1996**, *26*, 1075–1080, doi:10.1017/s003329170003539x.
127. Levitt, A.J.; Joffe, R.T.; King, E. Dim versus Bright Red (Light-Emitting Diode) Light in the Treatment of Seasonal Affective Disorder. *Acta Psychiatr. Scand.* **1994**, *89*, 341–345, doi:10.1111/j.1600-0447.1994.tb01526.x.
128. Martinez, B.; Kasper, S.; Ruhrmann, S.; Möller, H.J. Hypericum in the Treatment of Seasonal Affective Disorders. *J. Geriatr. Psychiatry Neurol.* **1994**, *7* (Suppl. S1), S29–S33, doi:10.1177/089198879400700109.

129. Eastman, C.I.; Lahmeyer, H.W.; Watell, L.G.; Good, G.D.; Young, M.A. A Placebo-Controlled Trial of Light Treatment for Winter Depression. *J. Affect. Disord.* **1992**, *26*, 211–221, doi:10.1016/0165-0327(92)90098-q.
130. Magnusson, A.; Kristbjarnarson, H. Treatment of Seasonal Affective Disorder with High-Intensity Light. A Phototherapy Study with an Icelandic Group of Patients. *J. Affect. Disord.* **1991**, *21*, 141–147, doi:10.1016/0165-0327(91)90061-v.
142. Chan, J.W.; Lam, S.P.; Li, S.X.; Chau, S.W.; Chan, S.Y.; Chan, N.Y.; Zhang, J.H.; Wing, Y.K. Adjunctive Bright Light Treatment with Gradual Advance in Unipolar Major Depressive Disorder with Evening Chronotype-A Randomized Controlled Trial. *Psychol. Med.* **2020**, 1–10, doi:10.1017/S0033291720003232.
143. Danilenko, K.V.; Lebedinskaia, M.Y.; Gadetskaia, E.V.; Markov, A.A.; Ivanova, Y.A.; Aftanas, L.I. A 6-Day Combined Wake and Light Therapy Trial for Unipolar Depression. *J. Affect. Disord.* **2019**, *259*, 355–361, doi:10.1016/j.jad.2019.08.051.
144. Yorguner Kupeli, N.; Bulut, N.S.; Carkaxhiu Bulut, G.; Kurt, E.; Kora, K. Efficacy of Bright Light Therapy in Bipolar Depression. *Psychiatry Res.* **2018**, *260*, 432–438, doi:10.1016/j.psychres.2017.12.020.
145. Sikkens, D.; Riemersma-Van der Lek, R.F.; Meesters, Y.; Schoevers, R.A.; Haarman, B.C.M. Combined Sleep Deprivation and Light Therapy: Clinical Treatment Outcomes in Patients with Complex Unipolar and Bipolar Depression. *J. Affect. Disord.* **2019**, *246*, 727–730, doi:10.1016/j.jad.2018.12.117.
146. Chojnacka, M.; Antosik-Wójcińska, A.Z.; Dominiak, M.; Bzinkowska, D.; Borzym, A.; Sokół-Szawłowska, M.; Bodzak-Opolska, G.; Antoniak, D.; Święcicki, Ł. A Sham-Controlled Randomized Trial of Adjunctive Light Therapy for Non-Seasonal Depression. *J. Affect. Disord.* **2016**, *203*, 1–8, doi:10.1016/j.jad.2016.05.062.
147. Lam, R.W.; Levitt, A.J.; Levitan, R.D.; Michalak, E.E.; Cheung, A.H.; Morehouse, R.; Ramasubbu, R.; Yatham, L.N.; Tam, E.M. Efficacy of Bright Light Treatment, Fluoxetine, and the Combination in Patients With Nonseasonal Major Depressive Disorder: A Randomized Clinical Trial. *JAMA Psychiatry* **2016**, *73*, 56–63, doi:10.1001/jamapsychiatry.2015.2235.
148. Suzuki, M.; Dallaspezia, S.; Locatelli, C.; Uchiyama, M.; Colombo, C.; Benedetti, F. Discrepancy between Subjective and Objective Severity as a Predictor of Response to Chronotherapeutics in Bipolar Depression. *J. Affect. Disord.* **2016**, *204*, 48–53, doi:10.1016/j.jad.2016.06.044.
149. Camardese, G.; Leone, B.; Serrani, R.; Walstra, C.; Di Nicola, M.; Della Marca, G.; Bria, P.; Janiri, L. Augmentation of Light Therapy in Difficult-to-Treat Depressed Patients: An Open-Label Trial in Both Unipolar and Bipolar Patients. *Neuropsychiatr. Dis. Treat.* **2015**, *11*, 2331–2338, doi:10.2147/NDT.S74861.
150. Benedetti, F.; Riccaboni, R.; Locatelli, C.; Poletti, S.; Dallaspezia, S.; Colombo, C. Rapid Treatment Response of Suicidal Symptoms to Lithium, Sleep Deprivation, and Light Therapy (Chronotherapeutics) in Drug-Resistant Bipolar Depression. *J. Clin. Psychiatry* **2014**, *75*, 133–140, doi:10.4088/JCP.13m08455.
151. Dauphinais, D.R.; Rosenthal, J.Z.; Terman, M.; DiFebo, H.M.; Tuggle, C.; Rosenthal, N.E. Controlled Trial of Safety and Efficacy of Bright Light Therapy vs. Negative Air Ions in Patients with Bipolar Depression. *Psychiatry Res.* **2012**, *196*, 57–61, doi:10.1016/j.psychres.2012.01.015.
152. Benedetti, F.; Dallaspezia, S.; Fulgosi, M.C.; Barbini, B.; Colombo, C.; Smeraldi, E. Phase Advance Is an Actimetric Correlate of Antidepressant Response to Sleep Deprivation and Light Therapy in Bipolar Depression. *Chronobiol. Int.* **2007**, *24*, 921–937, doi:10.1080/07420520701649455.
153. Martiny, K.; Lunde, M.; Undén, M.; Dam, H.; Bech, P. The Lack of Sustained Effect of Bright Light, after Discontinuation, in Non-Seasonal Major Depression. *Psychol. Med.* **2006**, *36*, 1247–1252, doi:10.1017/S0033291706008105.
154. Goel, N.; Terman, M.; Terman, J.S.; Macchi, M.M.; Stewart, J.W. Controlled Trial of Bright Light and Negative Air Ions for Chronic Depression. *Psychol. Med.* **2005**, *35*, 945–955, doi:10.1017/s0033291705005027.
155. McEnany, G.W.; Lee, K.A. Effects of Light Therapy on Sleep, Mood, and Temperature in Women with Nonseasonal Major Depression. *Issues Ment. Health Nurs.* **2005**, *26*, 781–794, doi:10.1080/01612840591008410.
156. Martiny, K. Adjunctive Bright Light in Non-Seasonal Major Depression. *Acta Psychiatr. Scand.* **2004**, *110*, 7–28, doi:10.1111/j.1600-0447.2004.00460_2.x.
157. Benedetti, F.; Colombo, C.; Pontiggia, A.; Bernasconi, A.; Florita, M.; Smeraldi, E. Morning Light Treatment Hastens the Antidepressant Effect of Citalopram: A Placebo-Controlled Trial. *J. Clin. Psychiatry* **2003**, *64*, 648–653, doi:10.4088/jcp.v64n0605.
158. Loving, R.T.; Kripke, D.F.; Shuchter, S.R. Bright Light Augments Antidepressant Effects of Medication and Wake Therapy. *Depress. Anxiety* **2002**, *16*, 1–3, doi:10.1002/da.10036.
159. Prasko, J.; Horacek, J.; Klaschka, J.; Kosova, J.; Ondrackova, I.; Sipek, J. Bright Light Therapy and/or Imipramine for Inpatients with Recurrent Non-Seasonal Depression. *Neuro Endocrinol. Lett.* **2002**, *23*, 109–113.
160. Beauchemin, K.M.; Hays, P. Phototherapy Is a Useful Adjunct in the Treatment of Depressed In-Patients. *Acta Psychiatr. Scand.* **1997**, *95*, 424–427, doi:10.1111/j.1600-0447.1997.tb09656.x.

161. Yamada, N.; Martin-Iverson, M.T.; Daimon, K.; Tsujimoto, T.; Takahashi, S. Clinical and Chronobiological Effects of Light Therapy on Nonseasonal Affective Disorders. *Biol. Psychiatry* **1995**, *37*, 866–873, doi:10.1016/0006-3223(94)00221-N.
162. Kripke, D.F.; Mullaney, D.J.; Klauber, M.R.; Risch, S.C.; Gillin, J.C. Controlled Trial of Bright Light for Nonseasonal Major Depressive Disorders. *Biol. Psychiatry* **1992**, *31*, 119–134, doi:10.1016/0006-3223(92)90199-a.
